# Antimetastatic Effects of *Phyllanthus* on Human Lung (A549) and Breast (MCF-7) Cancer Cell Lines

**DOI:** 10.1371/journal.pone.0020994

**Published:** 2011-06-16

**Authors:** Sau Har Lee, Indu Bala Jaganath, Seok Mui Wang, Shamala Devi Sekaran

**Affiliations:** 1 Department of Medical Microbiology, Faculty of Medicine, Universiti Malaya, Kuala Lumpur, Malaysia; 2 Biotechnology Centre, Malaysia Agricultural Research and Development Institute (MARDI), Serdang, Malaysia; Institute of Microbial Technology, India

## Abstract

**Background:**

Current chemotherapeutic drugs kill cancer cells mainly by inducing apoptosis. However, they become ineffective once cancer cell has the ability to metastasize, hence the poor prognosis and high mortality rate. Therefore, the purpose of this study was to evaluate the antimetastatic potential of *Phyllanthus* (*P. niruri*, *P. urinaria*, *P. watsonii*, and *P. amarus*) on lung and breast carcinoma cells.

**Methodology/Principal Findings:**

Cytotoxicity of *Phyllanthus* plant extracts were first screened using the MTS reduction assay. They were shown to inhibit MCF-7 (breast carcinoma) and A549 (lung carcinoma) cells growth with IC_50_ values ranging from 50–180 µg/ml and 65–470 µg/ml for methanolic and aqueous extracts respectively. In comparison, they have lower toxicity on normal cells with the cell viability percentage remaining above 50% when treated up to 1000 µg/ml for both extracts. After determining the non-toxic effective dose, several antimetastasis assays were carried out and *Phyllanthus* extracts were shown to effectively reduce invasion, migration, and adhesion of both MCF-7 and A549 cells in a dose-dependent manner, at concentrations ranging from 20–200 µg/ml for methanolic extracts and 50–500 µg/ml for aqueous extracts. This was followed by an evaluation of the possible modes of cell death that occurred along with the antimetastatic activity. *Phyllanthus* was shown to be capable of inducing apoptosis in conjunction with its antimetastastic action, with more than three fold increase of caspases-3 and -7, the presence of DNA-fragmentation and TUNEL-positive cells. The ability of *Phyllanthus* to exert antimetastatic activities is mostly associated to the presence of polyphenol compounds in its extracts.

**Conclusions/Significance:**

The presence of polyphenol compounds in the *Phyllanthus* plant is critically important in the inhibition of the invasion, migration, and adhesion of cancer cells, along with the involvement of apoptosis induction. Hence, *Phyllanthus* could be a valuable candidate in the treatment of metastatic cancers.

## Introduction

Tumors can be divided into two types; benign and malignant. Benign tumor is mainly localized and lacks the ability to spread to other parts of the body. Hence, they are rendered to be less harmful. On the other hand, malignant tumor which is more commonly known as cancer, had overcome the strict growth factors and adhesive requirements for their motility or metastatic ability [1]. Metastasis involves a series of complex processes governed by complicated mechanisms, beginning with the detachment of tumor cells, invasion, motility, adhesion to endothelial cells, and reestablishment of growth at a distant site [2]. Cells which are detached from the extracellular matrix often undergo apoptosis. Any resistance of these cells towards apoptosis will allow a successful metastatic dissemination. Cancer cells usually contain several mutations in the genes that regulate apoptotic process, therefore allowing them to evade programmed cell death. This superior resistance to apoptosis provides an advantage for the metastatic cells [3]–[6].

The metastasizing ability of malignant tumors is accountable for the poor prognosis and high mortality rate in cancer patients. Hence, metastasis is still a major clinical challenge for medical practitioners worldwide in cancer treatment [7]. Currently, there is still no absolute cure for cancer and its many devastating presentations [8]. Most cancers can be controlled by adopting appropriate conventional treatments such as surgery, radiation therapy and chemotherapy. However, these treatments have the potential to cause a range of side effects; hence the importance of conventional therapies may decline [9]. Alternative treatments founded in a ‘back-to-nature’ approach might yield improved treatment avenues with fewer or no undesirable side effects. In the search for these new treatments, natural products are carving a path as prospective anticancer agents.

The genus *Phyllanthus* is one of the most widely distributed plants throughout the Amazon rainforests as well as other tropical and subtropical regions. Numerous research studies on *Phyllanthus spp.* began in the late 1980's with the clinical efficacy of *Phyllanthus niruri* against viral Hepatitis B being observed [10]. *P. niruri*, locally known as “Dukung Anak” in Malaysia, is originated from India. *Phyllanthus* can now be found in almost every tropical countries due to its wide medicinal usages and lack of toxicity [10], [11]. Various therapeutic properties of this genus have been reported, including being antihepatotoxic, antilithic, antihypertensive, and most recently anti-HIV as well [4], [10]–[14]. There are also some speculation on the anticarcinogenic activity of various *Phyllanthus* plants. For example *P. emblica* has demonstrated growth inhibitory activity on A549 and HepG2 (liver carcinoma) [15], while the toxicity of *P. polyphyllus* on MCF-7, HT-29 (colon adenocarcinoma), and HepG2 was reported [16]. In another study, *Phyllanthus* was demonstrated to inhibit the growth of PC-3 (prostate adenocarcinoma) and MeWo (melanoma) via cell cycle arrest and apoptosis induction [17].

Until now, inhibition of cancer cell proliferation and induction of apoptosis have been thought as the markers to evaluate the effectiveness of anticancer drugs or cancer chemopreventive agents [18], [19]. Therefore, most of the currently available natural product-derived chemotherapeutic drugs kill cancer cells primarily by inducing apoptosis. Since malignancy of tumors is often attributed to their invasive and metastatic ability, a chemotherapeutic agent that only possesses the ability to induce apoptosis may not be useful against this type of tumors. Hence, the main objective of this study was to assess the antimetastatic activity of *Phyllanthus* plants on two cancer cell lines (MCF-7 and A549). Prior to that, cytotoxic effects of *Phyllanthus* on the cancer cells were thoroughly screened in order to choose a non-toxic effective dose. Subsequently, the relationship of this antimetastatic effect with probable involvement of apoptosis was investigated.

## Materials and Methods

### Plant extracts and Standard Drugs

The crude extracts (aqueous and methanolic) and their two fractions of each *Phyllanthus spp.*, namely *P. niruri*, *P. urinaria*, *P. watsonii* and *P. amarus*, were obtained from the Malaysian Agriculture and Research Development Institute (MARDI), Malaysia. The aqueous extracts and fractions were prepared by dissolving 10 mg in 1 ml of sterile PBS (Final concentration 10 mg/ml), whereas, the methanolic extracts were prepared by dissolving 40 mg in 1 ml of DMSO (Final concentration 40 mg/ml). The fractions were prepared by dissolving 10 mg in 1 ml of sterile PBS. The standard drugs used in this study were Cisplatin and Doxorubicin (MERCK). These standard drugs were prepared by dissolving 1 mg in 1 ml of sterile PBS to achieve a stock concentration of 1 mg/ml. The tubes containing the extracts and drugs were wrapped with aluminium foil and stored at −20°C until use. A single batch of extracts was used for all the experiments.

### Cell Culture

The cancer cell lines used in this study include human lung carcinoma (A549) and human breast carcinoma (MCF-7), whereas the normal cell lines used were human bronchus epithelium (NL20) and human breast epithelium (184B5). All cells were purchased from American Type Culture Collection (ATCC, USA). A549 and MCF-7 were grown in RPMI-1640 (Roswell Park Memorial Institute) and DMEM (Dulbecco's modified Eagles Medium) respectively while NL20 and 184B5 were grown in F-12K (ATCC, USA) and Mammary Epithelial Growth Medium (Lonza) respectively. To ensure growth and viability of the cells, the mediums were supplemented with 10% FBS (Gibco) and incubated in a humidified atmosphere with 5% CO_2_ at 37°C.

### High performance liquid chromatography (HPLC) coupled with Electron Spray Ionization (ESI) and Mass Spectrometry (LC-MS-MS) analysis

Supernatant of the aqueous extract sample was dried using a vacuum concentrator (concentrator 5301 eppendorf, Germany). For LC-MS-MS analysis, the lyophilized sample was redissolved into 20 mg/ml with 30% methanolic. Meanwhile, the total supernatant of the methanolic extract samples were evaporated using a rotary evaporator (rotavapor RII, BUCHI, Switzerland) and redissolved in 20% methanolic. The resulting products were then subjected to a solid phase extraction (SPE) column (LiChrolut RP-18 1000 mg/6 ml, Merck Germany) with mobile phase of 60% and 70% methanolic. All elutes collected were concentrated to 0.5 ml, and then diluted 8 times with 40% methanolic before the LC-MS-MS analysis was performed.

The HPLC system used consisted of a HPLC binary pump, diode array detector (DAD), and an auto-sampler injector compartment (1200 series, Agilent Technologies, Germany). For separation, C-18, 150 mm×4.6 mm i.d, 5 µm particle size Thermo Hypersil GOLD column (Thermo Scientific, UK) was chosen as the reverse phase while the mobile phase was 0.1% formic acid in water (solvent A) and 0.1% formic acid in acetonitrile (solvent B) with the gradient setting of solvent B: 5% (5 min), 5–90% (60 min), 5% (4 min) at a flow rate of 1 ml/min. Detection wavelengths were both set at 280 nm and 360 nm with constant injection volume at 20 µl. A 3200 QTrap LC/MS/MS system (Applied Bioscience – MDS Sciex) was used for the mass spectrometry analysis, with the iron source and voltage maintained at 500°C and −4.5 kV for negative ionization, respectively. The nitrogen generator was set at 60 psi curtain gas flow, 60 psi exhaust gas flow, and 90 psi source gas flow. The scanning modes chosen were Enhance Mass Spectrometer (EMS) and Enhance Ion Product (EIP) for full scan mass spectra that ranged from mass to charge ratio (*m/z*) of 100–1200.

### MTS (3-(4,5-dimethylthiazol-2-yl)-5(3-carboxymethoxyphenyl)-2-(4-sulfophenyl)-2H-tetrazolium) Cytotoxicity Assay

Cells were seeded at their optimal cell density (1×10^4^ cells/well) into a 96-well microtiter plate and were incubated overnight to allow cell attachment. They were then treated with *Phyllanthus* fractions as well as both the aqueous and methanolic *Phyllanthus* extracts at a 6-points serial dilution up to a final concentration of 1000 µg/ml. Vehicle-control wells with cells only and compound-control wells with extracts only were included. Plates were incubated at 37°C, 5% CO_2_ and 100% humidity for 24, 48, and 72 hours. At the end of each incubation period, the cell viability was determined using CellTiter 96® AQ_ueous_ Non-Radioactive Cell Proliferation Assay (Promega, USA) according to the manufacturer's instructions. Absorbance was measured using GloMax Multi Detection System (Promega, USA) at a wavelength of 490 nm with a reference wavelength of 750 nm. The respective Half Maximal Inhibitory Concentration (IC_50_) [20], [21] values at 72 hours incubation for individual plant extracts were determined and used in succeeding assays.

### Cell Invasion Assay

Cell invasion was determined using 24-well transwell chamber with 8 µm pore polycarbonate filter coated with basement membrane extracts (Cultrex, Trevigen). Cells were seeded into the upper compartment at a concentration of 2×10^6^ cells/ml in a volume of 100 µl/well. The lower compartment contained 500 µl of medium supplemented with extracts and 10% FBS as chemoattractants while serum-free medium was used as control. After incubation for 48 hours at 37°C, cells that had passed through the filters were detached using Cell Detachment Solution containing Calcein AM. Fluorescence intensity was measured with excitation wavelength at 485 nm and emission wavelength at 520 nm. Invasion inhibition rate was calculated using the following formula:

Invasion inhibition rate = (Mean of fluorescence of test wells−Mean of fluorescence of negative control wells)/(Mean of fluorescence of positive control wells−Mean of fluorescence of negative control wells)×100%.

### Scratch Motility Assay

Cells were seeded in a 24-well plate at 1×10^5^ cells/well and were allowed to grow overnight to reach confluency. The monolayer was then scratched with a pipette tip, washed with PBS twice to remove floating cells, and treated with extracts at their respective IC_50_ values. At the end of each incubations, the cells migrated into the scratched area was photographed and counted at 5 randomly selected field. The migrated cells were expressed as mean value per field.

### Cell Migration Assay

Transwell chambers (Millipore, Billerica, MA) were used in the cell migration experiments. Cells were seeded into the upper compartment of the transwell chamber at a concentration of 1×10^5^ cells/ml in a volume of 100 µl/well. Medium for the experimental and control groups were added into the lower compartment of the transwell chamber at 500 µl/well. At the end of incubation, cells that did not penetrate the polycarbonate membrane to the bottom of chamber were scraped off using a cotton sticker. The chamber plate was then placed onto a new 96-well feeder tray containing 150 µl of prewarmed Cell Detachment Solution in wells and incubated at 37°C for 30 minutes. One hundred microliters of 2× CyQuant NF (Invitrogen, Carlsbard, California) dye binding solution was then added into each well. Fluorescence intensity was measured with excitation wavelength at 485 nm and emission wavelength at 530 nm. Migration inhibition rate was calculated using the same formula as the invasion inhibition rate.

### Cell Attachment Assay

Cell attachment assay was carried out as described by Xia et al. with some modifications [22]. Briefly, cells treated with *Phyllanthus* extracts were detached using 0.5% trypsin-EDTA and plated back on a new culture plate. After each incubation periods of 4 to 24 hours, the cell attachment status and morphology was observed and photographs were captured.

### DNA Fragmentation Analysis

DNA fragmentation was carried out as described by Lin et al. with some modifications [23]. Briefly, five hundred microliters of 5×10^5^ treated cells were lysed in 55 µl DNA lysis buffer [1 M Tris-HCI (pH 8.0), 0.5 M EDTA, and 100% Triton X-100] and incubated at 4°C for 30 minutes. DNA was extracted from the supernatant with an equal volume of phenol/choloroform/isoamyl alcohol (25∶24∶1). Samples were spun and the upper aqueous layer transferred to a new tube, to which an equal volume of ice-cold 100% ethanol and 1/10 volume of 3 M sodium acetate (pH 5.2) were added and incubated at −20°C overnight. After spinning the sample, supernatant was decanted, pellet air dried, and then dissolved in deionized water-RNase solution [10 mg/ml RNase I] and incubated at 37°C for 30 minutes. Equal amounts of DNA (10 µg/well) were electrophoresed in 1.2% agarose gel impregnated with ethidium bromide at 5 V for the first 5 minutes and increased to 100 V for 1 hour. DNA fragments were then visualized using a UV transilluminator.

### Caspase assay

Cells were seeded, treated with extracts at their respective IC_50_ values, and incubated at 37°C, 5% CO_2_ and 100% humidity for 72 hours. Caspases activity was then determined using Caspase-Glo 3/7 Assay (Promega, USA) according to the manufacturer's instructions. Briefly, lyophilized Caspase-Glo 3/7 substrate was resuspended in its buffer and 100 µl of this reagent was added into each well. The contents of the wells were mixed gently and incubated at room temperature for 1 hour. Luminescence of each sample was measured using Glomax-Multi Detection System (Promega, USA).

### TUNEL assay

Terminal Deoxynucleotidyl-Transferase mediated dUTP Nick End Labelling (TUNEL) assay was performed using ApopTag® Plus Peroxidase *In Situ* Apoptosis Detection Kit (Chemicon® International). Briefly, 1×10^5^ cells were harvested, fixed in 1% paraformaldehyde in PBS, pH 7.4 and dried on a silanized microscope slide. The specimen was then post-fixed in pre-cooled ethanol/acetic acid (2∶1) and quenched in 3% hydrogen peroxidase in PBS. Excess liquid was tapped off before 75 µl/5 cm^2^ of equilibration buffer was immediately applied on the specimen. Next, 55 µl/5 cm^2^ of working strength terminal deoxynucleotidyl transferase (TdT) enzyme was added and incubated at 37°C for 1 hour. After incubation, the specimen was placed in a coplin jar containing working strength stop/wash buffer followed by an addition of 65 µl/5 cm^2^ of antidigoxigenin peroxidase conjugate. Specimen was washed in four changes of PBS, stained with 75 µl/5 cm^2^ of peroxidase substrate, counterstained in 0.5% (w/v) methyl green followed by several washes with distilled water, n-butanol, and xylene. Finally, the specimen was mounted under a glass coverslip in Permount fluid and observed under a light microscope (Olympus BX51) at a magnification power of 200×. Images were captured using Olympus U-CMAD3 at three fields per slide.

### Lactate Dehydrogenase (LDH) assay

LDH assay was performed using CytoTox-ONE® Homogeneous Membrane Integrity Assay purchased from Promega, USA. Cells were seeded, treated, and incubated for 72 hours. No-cell control, untreated cells control, and maximum LDH release control wells were included in each plate. At the end of incubation, lysis solution was added to positive wells and further incubated for 30 minutes to generate maximum LDH release. An equal volume of CytoTox-ONE® Reagent was added into each well and incubated at room temperature for 10 minutes with a subsequent addition of stop solution. Fluorescence was recorded with an excitation wavelength of 560 nm and an emission wavelength of 590 nm within 1 hour to avoid increased background fluorescence.

### Data Analysis

Results were expressed as the mean ± Standard Error Mean (SEM) of data obtained from three independent experiments. All data were analyzed using one way ANOVA, followed by Dunnett's test for pairwise comparison. *P*<0.05 was considered statistically significant for all tests.

## Results

### Polyphenols identification in *Phyllanthus spp.*

Tables 1 and 2 show the polyphenol compounds present in both methanolic and water-soluble extracts obtained from various species of *Phyllanthus* after analysis by HPLC coupled with photodiode array (PDA) and MS-MS detection. Twelve main compounds were identified based on their retention times, UV spectra, parent mass spectra and secondary fragmentation patterns. These compounds include gallic acid, galloylglucopyronside, digalloylglucopyronside, trigalloylglucopyronside, tetragalloylglucopyronside, corilagen, geraniin, rutin, quercetin glucoside, quercetin diglucoside, quercetin rhamnoside, and caffeolquinic acid.

**Table 1:** Polyphenol compounds detected in aqueous extracts of *Phyllanthus species*.

Compounds	Retention time	[M-H]m/z	MS-MS fragmentation	*Phyllanthus* species
Gallic acid	3.8	169	125,169	*P. amarus*, *P. niruri*, *P. urinaria*, *P. watsonii*
Galloylglucopyronside	2.8	331	125, 169, 211, 271	*P. amarus*, *P. niruri*, *P. urinaria*, *P. watsonii*
Corilagen	18.0	633	301, 125, 169	*P. amarus*, *P. niruri*, *P. urinaria*, *P. watsonii*
Geraniin	22.0	951	301, 125, 169, 463	*P. amarus*, *P. niruri*, *P. urinaria*, *P. watsonii*
Rutin	26.0	609	301, 179, 151	*P. amarus*, *P. niruri*, *P. urinaria*, *P. watsonii*
Quercetin glucoside	27.0	463	301, 179, 151	*P. amarus*, *P. niruri*, *P. urinaria*, *P. watsonii*
Caffeolquinic acid	23.0	353	191	*P. amarus*, *P. niruri*, *P. urinaria*, *P. watsonii*
Digalloylglucopyronside	15.0	483	125, 169, 211, 271, 313	*P. amarus*, *P. niruri*, *P. watsonii*
Quercetin rhamnoside	30.0	447	301, 151	*P. urinaria*, *P. watsonii*
Trigalloylglucopyronside	23.0	635	125, 169, 211, 271, 313, 465	*P. urinaria*

**Table 2:** Polyphenol compounds detected in methanolic extracts of *Phyllanthus species*.

Compounds	Retention time	[M-H]m/z	MS-MS fragmentation	*Phyllanthus* species
Geraniin	12.0	951	301, 125, 169, 463	*P. amarus*, *P. niruri*, *P. urinaria*, *P. watsonii*
Quercetin diglucoside	9.0	625	463, 301	*P. niruri*
Trigalloylglucopyronside	13.0	635	125, 169, 211, 271, 313, 465	*P. urinaria*
Tetragalloylglucopyronside	15.0	787	169, 211, 313, 465	*P. urinaria*

### Effect of *Phyllanthus* extracts, fractions and standard drugs on the growth and morphology of different cell lines

The MTS assay was used to investigate the potential cytotoxic effects of *Phyllanthus* extracts and fractions on different cell lines, where the cells were treated at increasing concentrations up to 1000 µg/ml for 24, 48, and 72 hours. Two standard drugs, namely Cisplatin and Doxorubicin were used as positive controls in this study, where the cells were treated at increasing concentrations up to 10 µg/ml. The cytotoxicity was recorded as IC_50_ (µg/ml) values, which resembles the concentration of extracts, fractions or drugs that kills or inhibits the growth of 50% of the population (Table 3). Data obtained showed that *Phyllanthus* extracts have the potential to inhibit growth of A549 and MCF-7 in a time- and dose-dependent manner with minimal effect on NL20 and 184B5. As a comparison, growth inhibition of *Phyllanthus* extracts on MCF-7 cells is shown to be more effective than on A549 cells. The data demonstrates that methanolic extracts of *Phyllanthus* exhibited greater cytotoxicity compared to the aqueous extracts for both cancer cell lines. Among the four *Phyllanthus* species, *P. watsonii* showed the strongest cytotoxicity with lowest IC_50_ values for both aqueous and methanolic extracts on A549 and MCF-7 respectively, followed by *P. urinaria*, *P. amarus*, and *P. niruri*. In contrast, fractions of *Phyllanthus* were not as effective as the *Phyllanthus* extracts. Fraction 1 showed very low toxicity to both cancer cell lines. Fraction 2 was more toxic to cancer cells compared to fraction 1, but not as toxic as *Phyllanthus* extracts as a whole. Fraction 2 also displayed toxicity to the normal cells. Both standard drugs showed strong cytotoxicity on A549 and MCF-7 cells with IC_50_ values <10 µg/ml as well. However, they were also very toxic to the normal cell lines with IC_50_ values comparable to their IC_50_ values for cancer cells.

**Table 3:** Cytotoxic effect [IC_50_ (µg/ml)] of *Phyllanthus* extracts and standard drugs, against two cancer (A549, MCF-7) and two normal cell lines (NL20, 184B5) after 72 hours incubation.

			IC_50_ (µg/ml) ± SEM			
			Cancer Cell Lines		Normal Cell Lines	
		Solvents	A549	MCF-7	NL20	184B5
Plant Extracts	*P. niruri (P.n)*	Aqueous	466.7±41.63	179.7±0.58	>500	>500
		Methanolic	128.3±17.56	62.3±9.07	>500	>500
	*P. urinaria (P.u)*	Aqueous	215.0±21.79	139.3±1.16	>500	>500
		Methanolic	69.0±11.53	48.7±10.02	>500	>500
	*P. watsonii (P.w)*	Aqueous	198.3±10.41	104.0±10.39	>500	>500
		Methanolic	61.3±16.17	49.0±8.19	>500	>500
	*P. amarus (P.a)*	Aqueous	240.0±26.46	156.7±5.77	>500	>500
		Methanolic	126.7±7.64	56.3±6.66	>500	>500
Standard Drugs	Cisplatin		7.6±1.10	1.4±0.54	0.9±0.05	3.0±0.03
	Doxorubicin		0.6±0.08	0.4±0.05	0.3±0.02	0.6±0.03
Fraction 1	*P. niruri (P.n)*	Aqueous	380.0±18.03	438.3±11.55	266.7±41.93	283.3±25.17
	*P. urinaria (P.u)*	Aqueous	>500	>500	>500	231.7±18.93
	*P. watsonii (P.w)*	Aqueous	395.0±8.66	376.7±2.89	241.7±20.21	230.0±26.46
	*P. amarus (P.a)*	Aqueous	>500	>500	>500	>500
Fraction 2	*P. niruri (P.n)*	Aqueous	228.3±5.77	81.7±16.07	108.3±5.77	230.0±50.74
	*P. urinaria (P.u)*	Aqueous	225.0±13.23	61.7±12.58	95.0±5.00	230.0±13.23
	*P. watsonii (P.w)*	Aqueous	225.0±43.30	46.7±10.41	105.0±5.00	201.7±20.21
	*P. amarus (P.a)*	Aqueous	264.3±45.24	70.0±17.32	106.7±7.64	213.3±54.85

Data is expressed as a mean of three independent experiments ± Standard Error Mean (SEM).

Upon treatment with *Phyllanthus* extracts and standard drugs for 72 hours, both A549 and MCF-7 cells displayed significant morphological changes. Observations showed that some cells were detached from the monolayer and some were rounded up. Furthermore, some of the cells had become granulated and vacuolated, possessed condensed chromatin, and displayed membrane blebbing.

In order to further evaluate the effects of *Phyllanthus* extracts on the metastatic activity of A549 and MCF-7, it was necessary to choose a non-toxic effective dose. From the toxicity testing, we obtained three sets of IC_50_ values at three different incubation time points (24, 48, and 72 hours). However, IC_50_ values at 72 hours incubation were chosen for subsequent experiments since the cells' viability remained above 80% when the cells were treated at this dose for 24 and 48 hours. The maximum incubation time for most of the antimetastatic assays was only up to 48 hours.

### Effect of *Phyllanthus* extracts on cell invasion

**Figure 1:**
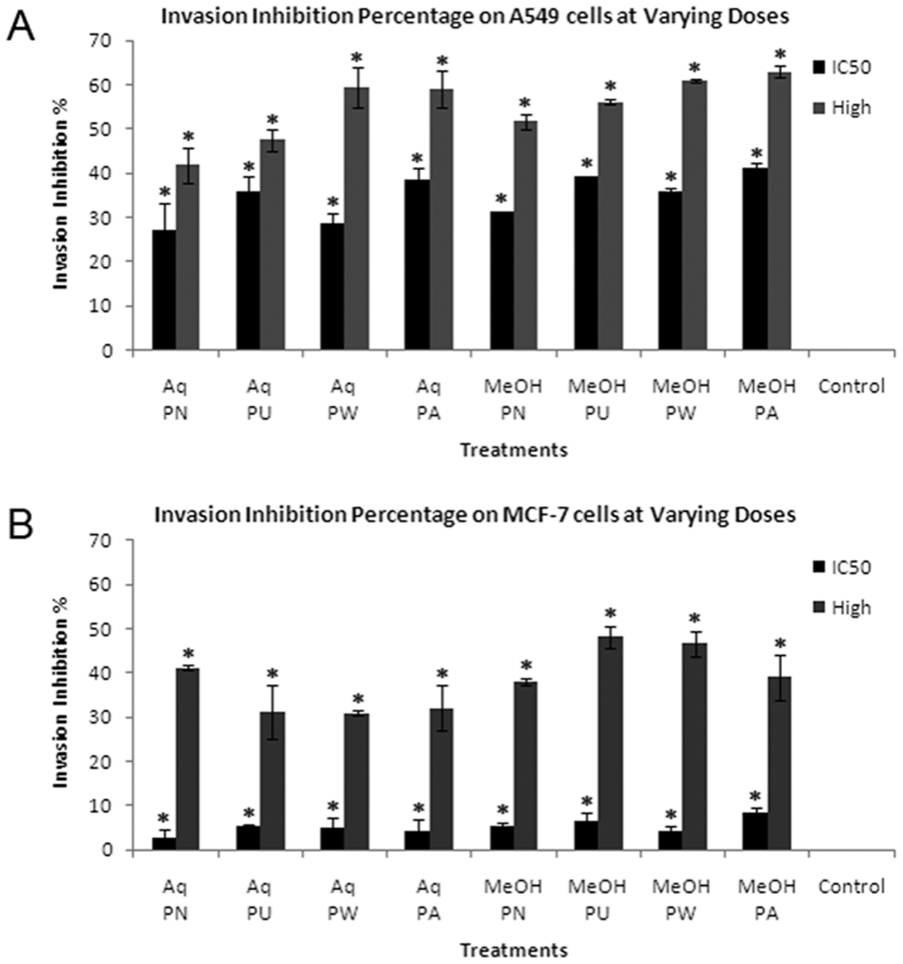
Invasion inhibition percentage of *Phyllanthus* on (A) A549 and (B) MCF-7 cells treated at varying concentrations. Error bar indicates the standard error of the mean of three independent experiments. PN – *P. niruri*, PU – *P. urinaria*, PW – *P. watsonii*, PA – *P. amarus*, Control – Non-treated cells. *P<0.05 vs control.

The antiinvasive effect of *Phyllanthus* was studied by using a transwell chamber coated with basement membrane extract that occludes the pores of the membrane. This is to avoid non-invasive cells from migrating through the membrane while allowing the invasive cells to detach themselves from surrounding cells and invade through the matrix in response to a chemoattractant. Therefore, the number of cells that were able to pass through the matrix and 8 µm pore into the lower well emulates their invasive potential. As shown in Figure 1, the *Phyllanthus* extracts prevent the invasion of both A549 and MCF-7 cells in a dose-dependent manner (*P*<0.05). When the cells were treated with extracts at their respective IC_50_ concentrations, they inhibit invasion of A549 cells (20%–40%) more strongly as compared to MCF-7 cells (less than 10%). At higher doses however (200 µg/ml for methanolic extracts and 500 µg/ml for aqueous extracts), both cancer cells' invasion was inhibited to a greater extent (40%–60% for A549 cells and 30%–50% for MCF-7 cells).

### Effect of *Phyllanthus* extracts on cell migration

**Figure 2:**
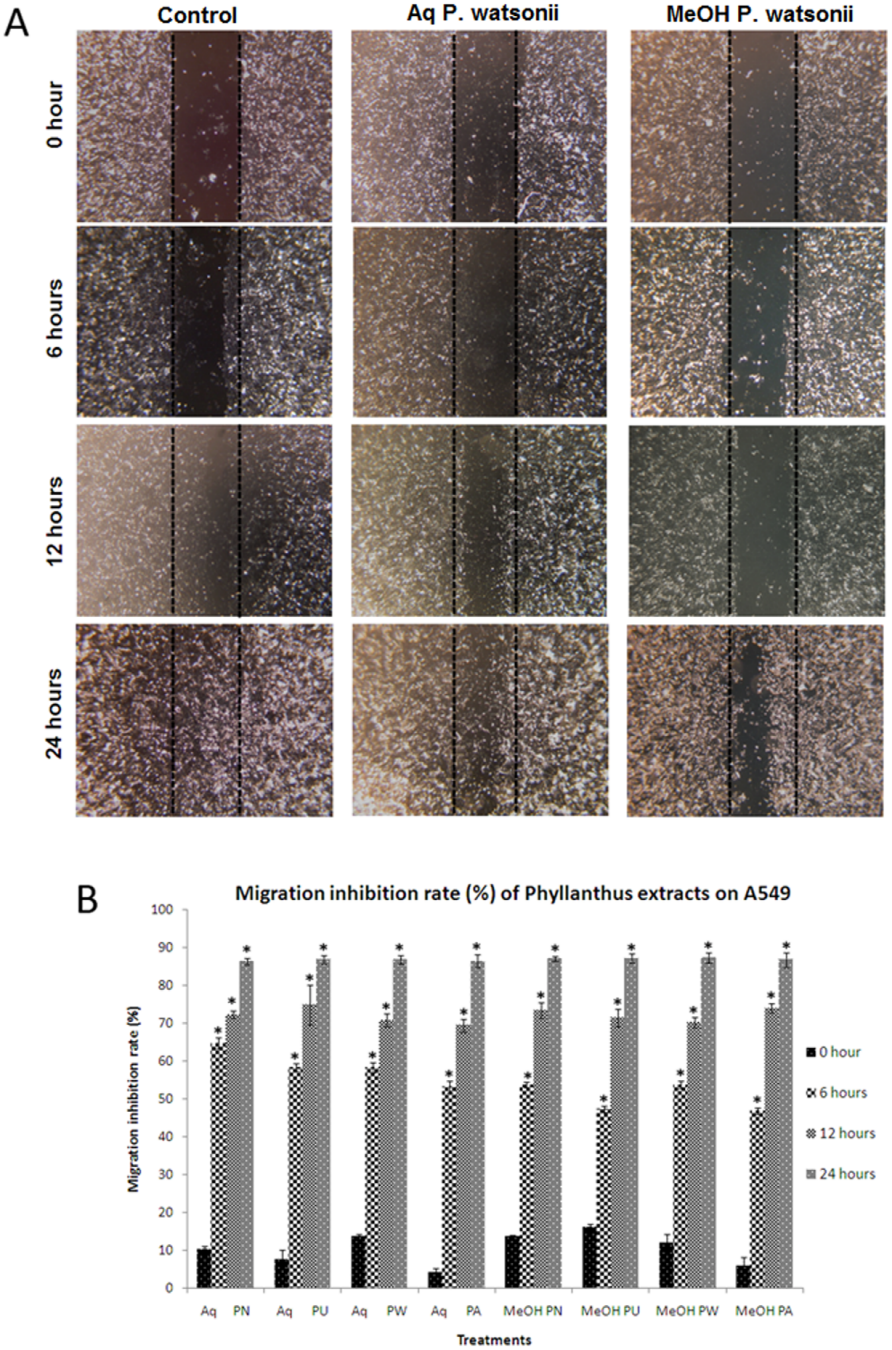
Wound closure activity of treated A549 cells after 24 hours. (A) Representative photographs of wounded A549 cell monolayer treated with aqueous and methanolic *P. watsonii* extracts. Typical result from three independent experiments is shown. (Magnification power: 200×) (B) Quantitative assessment of migration inhibition rate of *Phyllanthus* on A549 cells. Error bar indicates the standard error of the mean of three independent experiments. PN – *P. niruri*, PU – *P. urinaria*, PW – *P. watsonii*, PA – *P. amarus*. *P<0.05 vs control.

**Figure 3:**
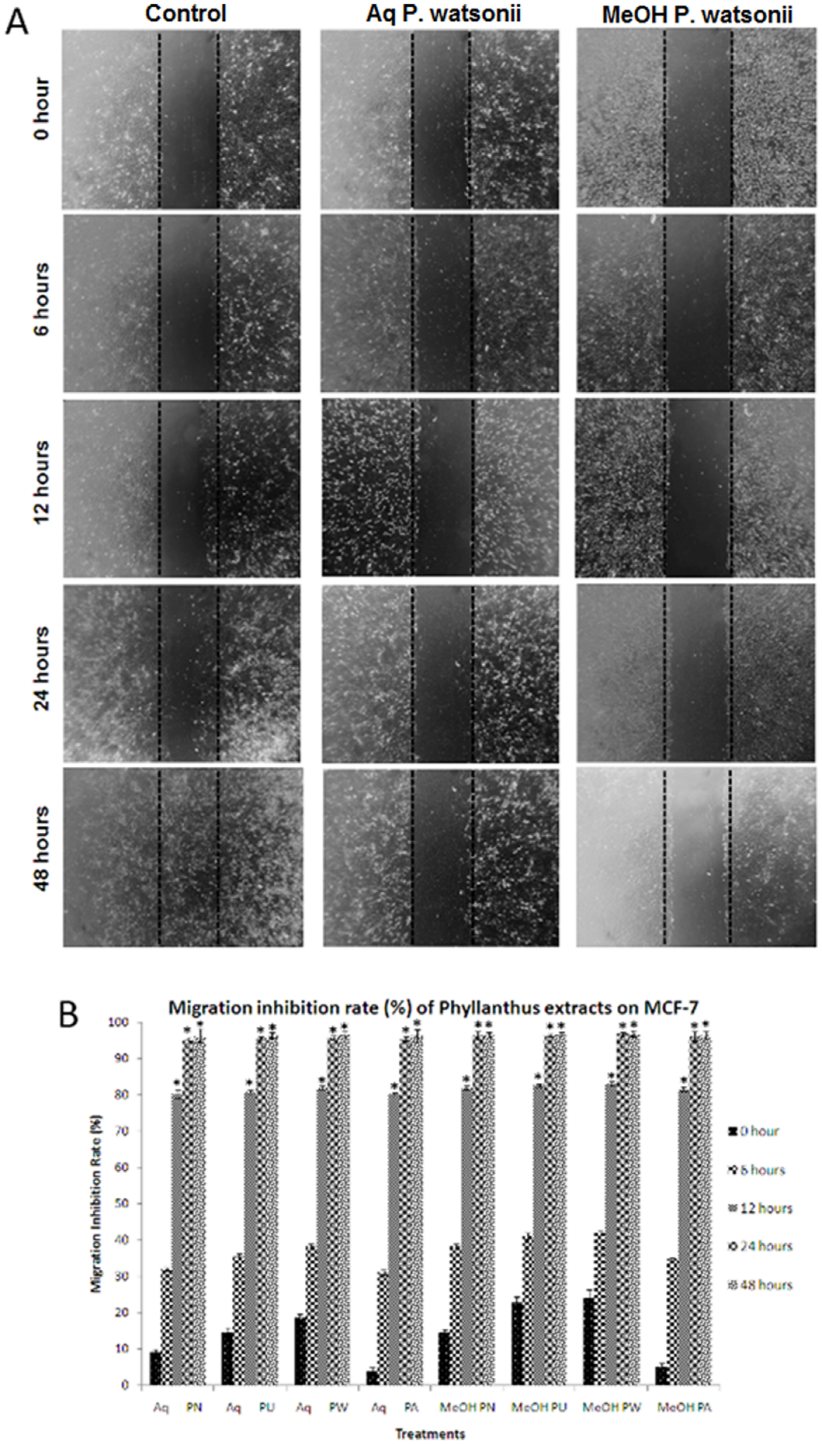
Wound closure activity of treated MCF-7 cells after 24 hours. (A) Representative photographs of wounded MCF-7 cell monolayer treated with aqueous and methanolic *P. watsonii* extracts. Typical result from three independent experiments is shown. (Magnification power: 200×) (B) Quantitative assessment of migration inhibition rate of *Phyllanthus* on MCF-7 cells. Error bar indicates the standard error of the mean of three independent experiments. PN – *P. niruri*, PU – *P. urinaria*, PW – *P. watsonii*, PA – *P. amarus*. *P<0.05 vs control.

**Figure 4:**
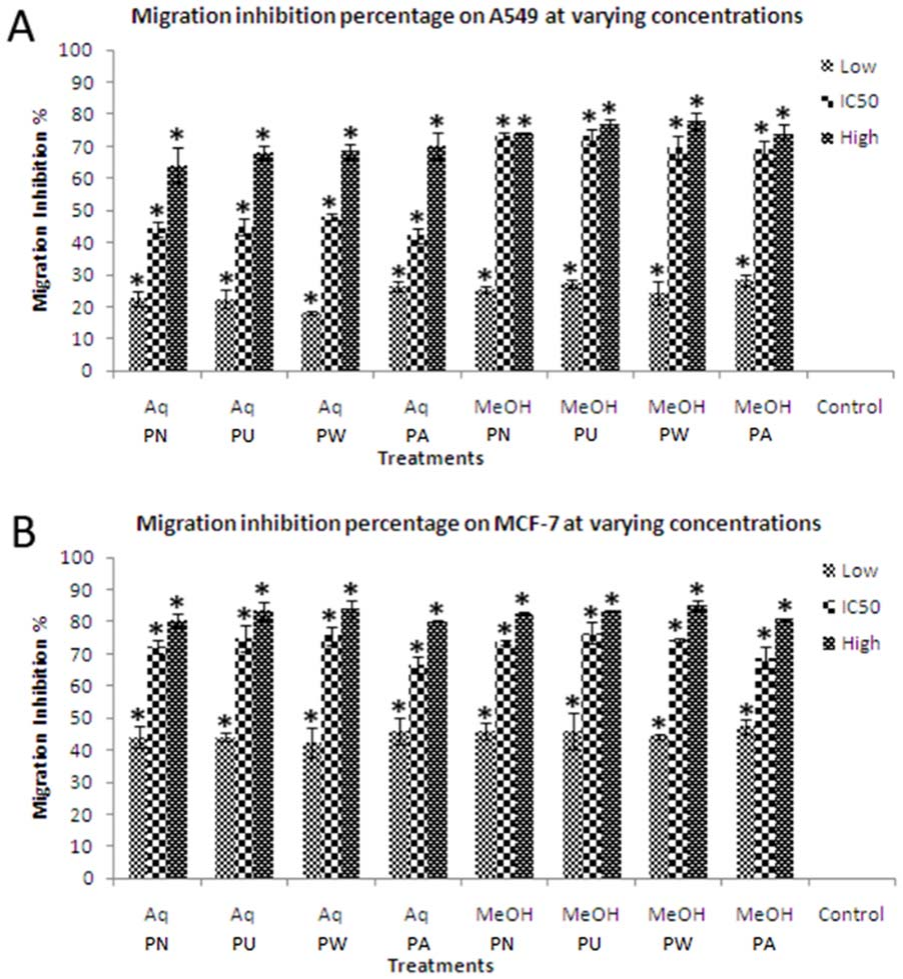
Migration inhibition percentage of *Phyllanthus* on (A) A549 and (B) MCF-7 cells treated at varying concentrations. Error bar indicates the standard error of the mean of three independent experiments. PN – *P. niruri*, PU – *P. urinaria*, PW – *P. watsonii*, PA – *P. amarus*, Control – Non-treated cells. *P<0.05 vs control.

The effect of *Phyllanthus* on A549 and MCF-7 cell migration was determined by scratch motility and cell migration assays. In the scratch motility assay, both the untreated A549 and MCF-7 cells exhibited a complete wound closure activity after treatment for 24 and 48 hours respectively (Figure 2A and 3A). In contrary, the *Phyllanthus*-treated cells showed only a limited closure of wound at the end of their respective incubation time by forming asymmetric lamellipodial protrusions into the denuded zone. The number of migrated cells were calculated from five randomly selected fields per sample and represented as migration inhibition rate in Figure 2B and 3B. As the incubation time increased, the migration inhibition rate also significantly increased (*P*<0.05). Additionally, *Phyllanthus* showed a reduction in migration of A549 and MCF-7 cells in a dose-dependent manner using the cell migration assay (Figure 4). The migration of cells was significantly decreased (*P*<0.05) when treated with methanolic extracts (20–200 µg/ml) and aqueous extracts (50–500 µg/ml). Even at the lowest concentration tested, migration inhibition exerted by *Phyllanthus* was greater than 20% for A549 cells and greater than 40% for MCF-7 cells.

### Effect of *Phyllanthus* extracts on cell adhesion

**Figure 5:**
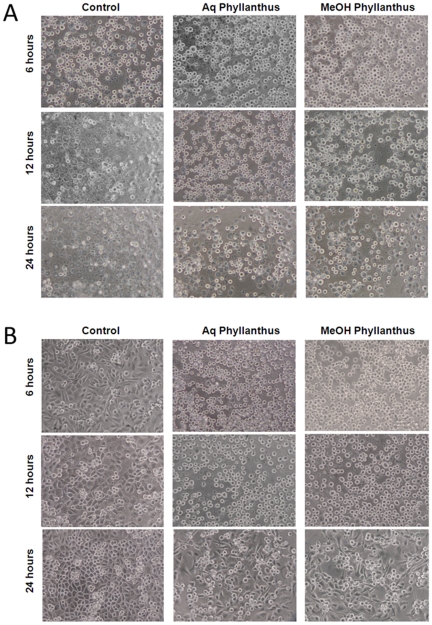
Cell adhesion status of treated (A) A549 and (B) MCF-7 cells after 24 hours incubation. Typical result from three independent experiments is shown. (Magnification power: 200×).

The effect of *Phyllanthus* on cell adhesion was examined by detaching the treated cells from the cultured flasks and plating them onto a new culture plate with the same number of viable treated cells in each group. The rounded cells represent the unattached cells, but all cells ultimately will attach themselves to the plate. Therefore, the higher number of rounded (unattached) cells at a given time point as compared to the untreated control signifies a delay or defect in their attachment. Figure 5 shows the different attachment ability between the *Phyllanthus*-treated and the untreated control cells. As can be seen from that figure, most of the untreated cells (both A549 and MCF-7) have begun to adhere to the plate after 6 hours of incubation. They even formed a monolayer after incubation for 24 hours. In contrast, the treated cells remained in their suspension form after incubation for 6 hours and began to adhere slightly only after incubation for 12 hours. Twenty-four hours later, some of the treated cells were still unattached, hence indicating that the adhesive capability of the *Phyllanthus*-treated cells was retarded.

### Effect of *Phyllanthus* extracts on caspase-3 and -7 activities

**Figure 6:**
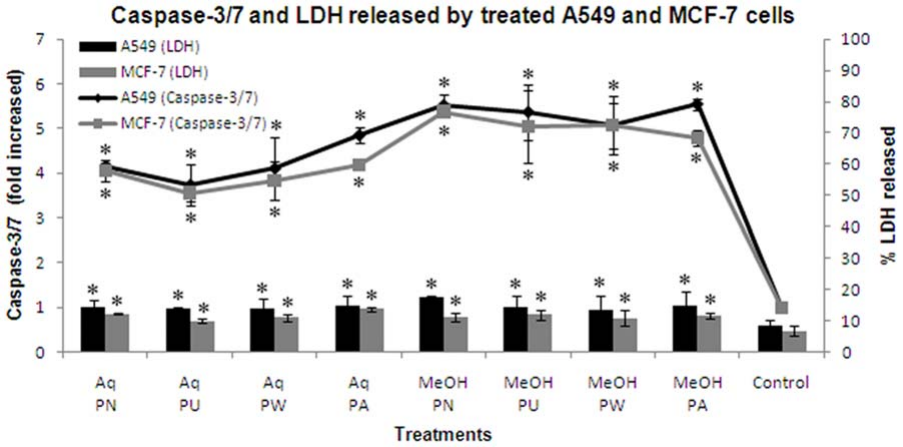
Caspase-3/7 and Lactate Dehydrogenase (LDH) level released from treated A549 and MCF-7 cells. Error bar indicates the standard error of the mean of three independent experiments. PN – *P. niruri*, PU – *P. urinaria*, PW – *P. watsonii*, PA – *P. amarus*, Control – Non-treated cells. *P<0.05 vs control.

Caspase-3 and -7 play crucial roles as early apoptosis biochemical markers in mammalian cells. Caspase-Glo® 3/7 Assay uses a luminogenic substrate containing the DEVD sequence which is selective for caspase-3 and -7. The caspases activity in both non-treated and *Phyllanthus*-treated cancer cells were measured after 72 hours and are shown in Figure 6. Caspases activity level detected in non-treated control cells corresponded to the portion of apoptotic cells present in the naturally growing population due to natural aging. In treated cells, activities of Caspase-3 and -7 increased from 3-fold to 5-fold (*P*<0.05) over basal levels signifying an activation of these caspases in both A549 and MCF-7 cells treated with *Phyllanthus* extracts.

### Effect of *Phyllanthus* extracts on cellular membrane integrity

Lactate Dehydrogenase (LDH) is a cytosolic enzyme released into cell culture supernatant due to compromised membrane integrity, which is associated with necrotic cell death. The extent of its activity in converting tetrazolium salt into red formazan product is proportional to the number of necrotic cells [24]. Figure 6 shows the percentage of LDH released from the A549 and MCF-7 cells after 72 hours of treatment with each aqueous and methanolic extracts of *Phyllanthus*. From the data, the LDH amount released by *Phyllanthus*-treated cells remained at low levels (<20%) comparable to the level of the untreated cells (<10%). Therefore, it suggests that *Phyllanthus* induces minimal cytotoxicity by disrupting membrane integrity which leads to necrosis.

### Effect of *Phyllanthus* extracts on nuclear fragmentation

**Figure 7:**
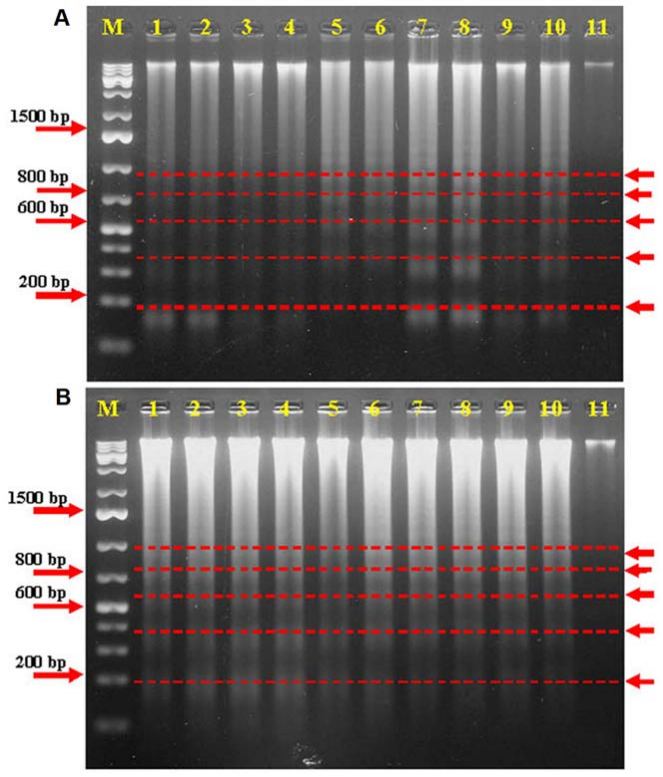
DNA fragmentation of (A) A549 and (B) MCF-7 cells treated with *Phyllanthus* and standard drugs. Red arrows at the right are pointing to the bands of DNA fragments. Typical result from three independent experiments is shown. M: Molecular-weight marker, Lane 1–4: aqueous extracts of *P. niruri*, *P. urinaria*, *P. watsonii*, and *P. amarus*, Lane 5–8: methanolic extracts of *P. niruri*, *P. urinaria*, *P. watsonii*, and *P. amarus*, Lane 9: Cisplatin, Lane 10: Doxorubicin, and Lane 11: Untreated control.

DNA fragmentation in condensed chromatin and formation of apoptotic bodies are some of the events of late apoptosis [25]. In order to further demonstrate the ability of *Phyllanthus* extracts to induce apoptosis, DNA fragmentation assay was used to verify the presence of apoptotic cells in the treated cultured cells. Figure 7 shows a typical ladder-like pattern of multiple, approximately 180–200 bps DNA fragments after A549 and MCF-7 cells were treated with the extracts, one of the hallmarks of apoptosis.

**Figure 8:**
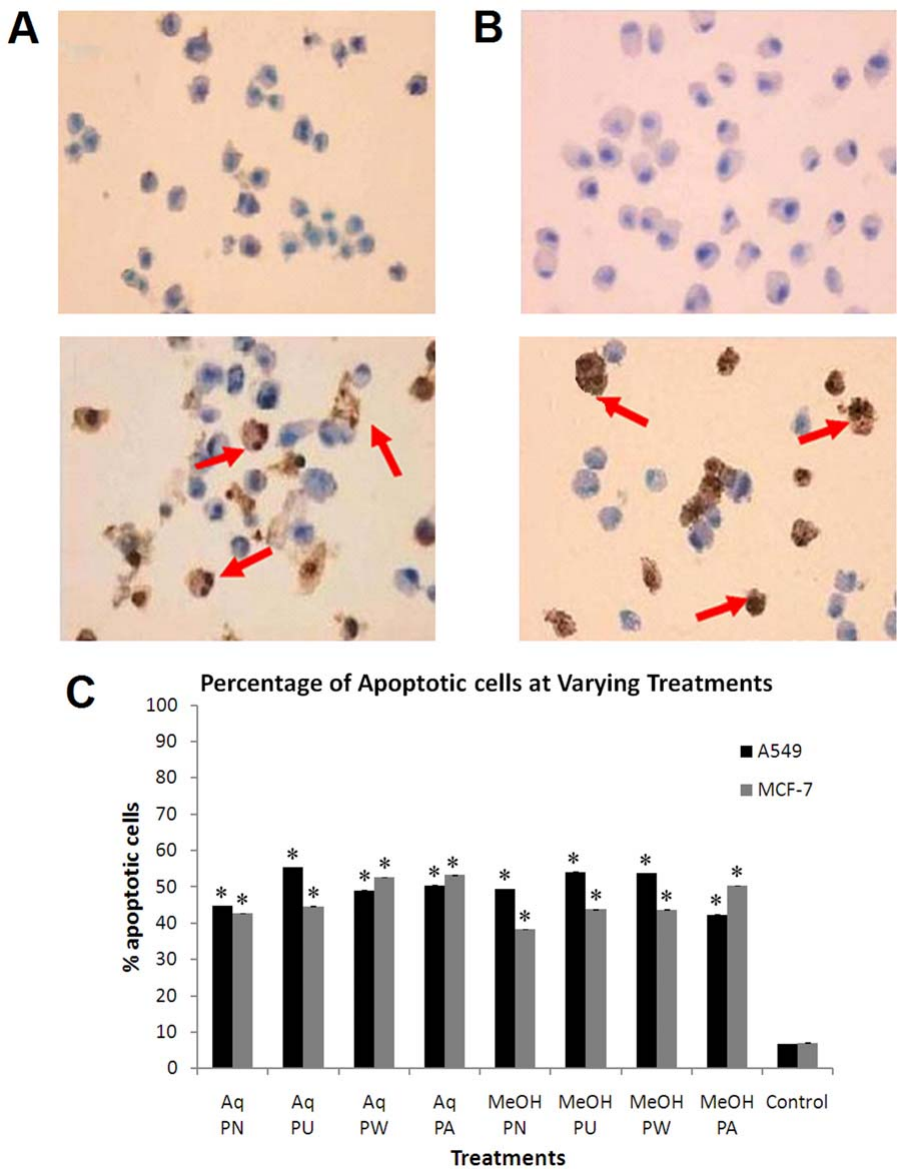
Red arrows showing TUNEL-positive (A) A549 cells (B) MCF-7 cells. Typical result from three independent experiments is shown. (Magnification power: 200×) (C) Quantitative assessment of apoptotic cells percentage of A549 and MCF-7 cells treated with *Phyllanthus*. Error bar indicates the standard error of the mean of three independent experiments. PN – *P. niruri*, PU – *P. urinaria*, PW – *P. watsonii*, PA – *P. amarus*, Control – Non-treated cells. *P<0.05 vs control.

Additionally, cell apoptosis was determined *in situ* based on the enzymatic labelling of free 3′-OH terminus of non-random DNA single-stranded and double-stranded breaks with modified nucleotides, resulting in the brown staining of the apoptotic cells. After 72 hours of treatment with *Phyllanthus* extracts, the percentage of apoptotic cells in both A549 and MCF-7, increased tremendously as compared to the untreated control cells. Since the cells were treated with the IC_50_ concentrations of extracts, the mean percentage of apoptotic cells observed from 3 views per slide varied from 38% up to 55%. Figure 8A and 8B shows the TUNEL-positive cells after treatment with aqueous and methanolic extracts of *Phyllanthus*. Meanwhile, the percentages of apoptotic cells for the effects of each individual *Phyllanthus* extracts on A549 and MCF-7 were illustrated in Figure 8C.

## Discussion

Over the years, the plants of the genus *Phyllanthus* from the family *Euphorbiaceae*, have gained reputation in folk and traditional medicine including ayurveda, siddha and traditional Chinese medicine for their myriad of healing properties. In this study, we aimed to investigate the antimetastatic activity of *Phyllanthus* on cancer cells. Prior to that, an effective dose which is non-toxic to the cells had to be determined. Hence, we initially evaluated the toxicity of both aqueous and methanolic extracts of four different species of *Phyllanthus* plants, namely *P. niruri*, *P. urinaria*, *P. watsonii*, and *P. amarus*, on two human cancer cell lines (A549 and MCF-7) and two normal human cell lines (184B5 and NL20). Our data showed that *Phyllanthus* exhibited selective cytotoxicity against MCF-7 and A549 human cancer cells, with IC_50_ values ranging from 50 µg/ml to 180 µg/ml and 65 µg/ml to 470 µg/ml, respectively for both methanolic and aqueous extracts while having minimal toxicity to the normal cell lines at the same concentrations. The determined IC_50_ doses were then used as the treatment condition for the subsequent experiments. In addition to that, the effects of two *Phyllanthus* fractions were also tested on both cancer and normal cell lines. The first fraction was non toxic or showed very little toxicity to cancer cells while the second fraction showed reduced toxicity to cancer cells but were toxic to normal cells. Sun and Liu reported that not any individual class of components in an extract could be entirely held accountable for the activity produced by the whole extract itself [18]. Therefore, it is more meaningful as well as prudent to assess the activity of *Phyllanthus* as a complete mixture of interacting bioactive compounds rather than evaluating them as a breakup of their individual components. The cytotoxic activities exhibited by natural products are mainly attributed to the presence of different bioactive compounds within the plant extracts [26]–[28]. HPLC analysis revealed the presence of various polyphenol compounds in *Phyllanthus* extracts as shown in Table 1 and 2. The polyphenol compounds identified can be broadly classified into four categories; ellagitannins, gallotannins, flavonoids, and phenolic acids [17]. Different species of *Phyllanthus* plants have a variation in the composition percentages of each bioactive component, thus giving rise to the different extent of cytotoxicity to cancer cells. Among the four *Phyllanthus* species studied, *P. watsonii* exhibited the highest cytotoxicity to both A549 and MCF-7 cells *in vitro*.

Ineffectiveness of current available treatments is mainly due to the invasive and metastatic properties of malignant cancer cells. The crucial factor that affects the invasion and metastasis of tumor is the integrity of the basement membrane that holds the tumor cells together [29]. Metastasis most often begins with tumor invasion which is correlated with the destruction of the extracellular matrix (ECM) and the basement membrane components by a synergistic action of a number of proteolytic enzymes including the matrix metalloproteinases (MMPs) [30]. The MMPs are a family of highly homologous, zinc- and calcium-dependent endopeptidases [31]–[32]. A genomic study done by Puente et al. discovered 24 distinct genes which encode for various MMPs [33], where MMP-2 and MMP-9 were deeply associated with cancer invasion and metastasis. This is because their elevated expression has increased the metastatic potential of tumor cells and they had also been known to be able to degrade type IV collagen-rich basement membrane of vessel wall [32], [34]. From our data, *Phyllanthus*-treated cells exhibited greater difficulties to invade the extracellular matrix as compared to the untreated cells, hence suggesting the ability of *Phyllanthus* to inhibit the production of MMPs, and thus limiting the invasive and metastatic capabilities of tumor cells. Inhibition of MMP expression could be due to the blockage of the Ras/Rho/MAP Kinase pathway since inhibition of Ras *in vitro* has been shown to stop MMPs production [35]. Additionally, inhibition of NF-κB and PI3K/AKT pathways could also lead to the inhibition of MMP expression [36]. However, clarification of the exact antiinvasion property of *Phyllanthus* against cancer cells warrants further studies.

Cell motility and adhesion are the next critical processes in metastasis upon the successful invasion of tumor cells into the blood or lymph capillaries. Since the lungs are the first organ that the detached tumor cells come upon most frequently, they become the main location for tumor metastasis [37]. Data obtained from cell migration assays displayed that *Phyllanthus* has the ability to stop migration of cancer cells. It can be argued that the reduction in cellular migration could be due to the cytotoxic effect exerted by *Phyllanthus* at high concentrations. However, a significant decrease of cellular mobility was also observed at IC_50_ and lower concentrations in which there were minimal cell death, indicating its ability to suppress and limit cells' motility. Once the tumor cell is arrested at a particular organ, it must be able to adhere strongly before it can colonize and establish a secondary tumor at the new site. In this study, we showed that the *Phyllanthus*-treated cells had a diminished capacity to attach at a new location compared to the untreated cells. This ability of *Phyllanthus* to inhibit cell's motility and adhesion can be correlated with its ability to inhibit the invasiveness of cells since inhibition of MMP-2 and MMP-9 activities had also been shown to be capable of reducing cells' migration [32], [38]. Besides that, *Phyllanthus* might also be affecting integrins, which are a family of transmembrane glycoproteins involved in various aspects of cell adhesion and migration. A recent study performed by Lee et al. showed that inhibition of integrin sialylation could inhibit cell adhesion and migration [39].

In the cell invasion and cell motility assays, cells unaffected by the extracts invaded and migrated to the lower chamber while the remaining cells were left at the top of the chamber. Similarly, in cell adhesion assay, not all the treated cells were able to reattach themselves. Hence, we were interested to further investigate how *Phyllanthus* had caused the cells to lose their normal ability to metastasize, where our main hypothesis was that the cancer cells were dying due to *Phyllanthus*. A disseminating tumor cell faces the possibility of losing its viability during anytime throughout the metastasis process [40]. They could have died simply due to mechanical destruction during the invasion or migration process (necrosis), or by *Phyllanthus* triggered cell death (apoptosis or necrosis). Apoptosis typically involves a series of events [24], beginning with the release of cytochrome c from mitochondria, activation of a cascade of caspases, degradation of poly ADP-ribose polymerase (PARP), and finally the fragmentation of chromosomal DNA [5], [41]. As opposed to apoptosis, necrosis is usually associated with external damage leading to accidental cell death, resulting in mitochondrial and cytoplasmic swelling, followed by compromised membrane integrity that will eventually burst, releasing its cytoplasmic contents [25], [42].

Apoptosis can be divided into two pathways, the extrinsic and intrinsic pathways which involve caspases that are constitutively expressed during the process. Regardless of which pathway is initiated, both will eventually converge and activate Caspase-3 and -7, which are the execution caspases [43]. Based on our data, apoptosis occurred in the cells treated with *Phyllanthus* as the level of these execution caspases was increased manifold over the basal level of untreated cells. This could be due to the presence of tannins (such as gallic acid and geraniin) in the *Phyllanthus* extracts which had been shown to be able to induce apoptosis in several human cancer cells [4], [44]. Activation of caspase-3 will subsequently trigger the proteolytic cleavage of poly ADP-ribose polymerase resulting in DNA fragmentation that usually occurs during late apoptosis [5], [45]. These DNA fragments appear as DNA ladder on an agarose gel as shown in Figure 7 instead of a randomized DNA breakdown which is observed as a smear for necrosis. However, internucleosomal DNA fragmentation is not universal as it may not always occur during apoptosis [46]. But further *in situ* staining of the DNA breaks confirmed the induction of apoptosis by *Phyllanthus* with the presence of TUNEL-positive cells as shown in Figure 8. Although the data obtained suggest apoptosis as the mode of cell death, the complex phytochemical mixture of *Phyllanthus* species allows a possibility of necrosis as the other mechanism of action. Some of the cytotoxic agents have the ability to activate both apoptotic and necrotic cell death pathways [42]. In addition, cells might also have died via necrosis as they invade or migrate through the membrane pores. Therefore, in order to differentiate between the dominant modes of cell death, release of LDH which is an indicator of necrosis, was assessed in *Phyllanthus*-treated cells. Our data revealed that LDH levels released in *Phyllanthus*-treated cells remained low. Therefore, the possibility of necrosis as the mode of cell death can be excluded.

Although the exact bioactive compound(s) in *Phyllanthus* exerting the antimetastasis effects are not yet identified, but it is already known that *Phyllanthus* is abundant in flavonoids, phenolic acids and ellagitannins. Many of these bioactive compounds have been shown to exert antimetastatic and apoptosis-inducing effects. For instance, gallic acid prevents the metastasis of AGS and U87 cells via inhibition of NF-κB activity, suppression of metalloproteinases activities, as well as downregulation of Ras/PI3K/AKT and Ras/MAPK signaling pathways [36], [47]. In addition, flavones and plant polyphenols have been shown to exert antimetastatic and antiinvasion activities by inhibiting matrix-degrading proteases [30], [32]. Another phenolic compound, 5-caffeoylquinic acid isolated from *Euonymus alatus* has also been shown to be a strong MMP-9 inhibitor [34]. Thus, there is a possibility that the presence of flavonoids/phenolic acids/ellagitannins in *Phyllanthus* may have a crucial role in its antimetastatic actions.

In conclusion, our observations indicated that *Phyllanthus* were able to cause selective toxicity on A549 and MCF-7 cancer cell lines. In addition, it has the ability to exert inhibitory effects on the critical steps in metastasis, including cell invasion, migration, and invasion. Its antimetastatic potential could partially be attributed to its capability to induce apoptosis which is associated with the activation of caspases-3 and -7 as well as DNA fragmentation. As evidenced from the above results, *Phyllanthus* might be an important candidate as a chemopreventive agent against cancer metastasis. Nevertheless, a better understanding on the exact mechanisms of how *Phyllanthus* exerts its antimetastatic activity would be essential and hence, further investigations are needed.
